# Intensity modulated radiotherapy (IMRT) in the management of locally advanced oropharyngeal squamous cell carcinomata (SCC): disease control and functional outcome using the therapy outcome measure (TOM) score - report from a single U.K. institution

**DOI:** 10.1186/1758-3284-2-28

**Published:** 2010-10-14

**Authors:** Charlotte J Ingle, Kent Yip, Valerie Caskie, Catherine Dyson, Amanda Ford, Christopher D Scrase

**Affiliations:** 1 Department of Clinical Oncology, Ipswich Hospital NHS Trust, Suffolk, U.K; 2 Department of Speech and Language Therapy, Ipswich Hospital NHS Trust, Suffolk, U.K; 3 Department of Dietetics, Ipswich Hospital NHS Trust, Suffolk, U.K

## Abstract

**Introduction:**

This paper evaluates tumour control and toxicity especially in relation to swallowing dysfunction in those patients with locally advanced oropharyngeal squamous cell carcinoma who have undergone either primary chemo-radiation or post-operative parotid sparing IMRT. The TOM scoring system was used to assess dysphagia.

**Methods:**

All patients with locally advanced (stage 3/4) squamous cell oropharyngeal cancer and who required either primary or post-operative RT were identified. Toxicity was recorded prospectively. The TOM score (0-5 where 5 indicates that the patient is able to eat a normal diet and 0-2 varying degrees of enteral feeding dependency), weights and trismus was recorded immediately prior to and following radiotherapy.

**Results:**

24 patients were identified between 1/2003 and 11/2007. Median weight loss during radiotherapy was 9 kg. All but one patient had a gastrostomy (RIG) tube inserted prophylactically. With a mean follow-up of 37.1 months, 62.5% of pts had a TOM score of 5, 12.5% scored 3, 8% scored and 17% scored 0-2.. For those patients whose swallowing function did recover, it took on average 8.7 months. 15% patients experienced trismus secondary to radiotherapy. 2 year overall survival was 92% and disease specific survival 96%.

**Conclusion:**

Excellent disease control with intensified schedules of radiotherapy with IMRT has been achieved in this patient population. Intermediate toxicity is significant but with longer follow-up, dysphagia continues to improve with 75% of patients not requiring any form of enteral or oral supplementation.

## Introduction

Squamous cell carcinoma accounts for about 90% of new head and neck cancers. There are on average 8000 new cases per year and 3000 deaths in England and Wales alone (Cancer Research UK). Risk factors for the development of squamous cell carcinoma of the head and neck (SCCHN), which is the commonest histological variant, include smoking, alcohol and previous radiotherapy in that locality. Human papilloma virus (HPV) is an independent risk factor for oropharyngeal carcinoma, in particular HPV-16 subtype and seropositivity for the oncoproteins E6 and E7 [1].

The treatment of oropharyngeal carcinoma consists primarily of surgery, radiotherapy, chemotherapy or a combination. There is no single therapeutic regimen that gives a clear-cut superior survival advantage over another. Treatment choice will be dependent on a number of patient- and treatment-related factors.

Depending on the subsite, early tumours of the oropharynx may be considered for surgery or irradiation whereas more locally advanced tumours require a multimodal approach with either surgery and adjuvant radiotherapy, or chemoradiotherapy with salvage surgery [2]. However, surgery for locally advanced tumours may result in significant morbidity.

Radiotherapy for both early and advanced base of tongue tumours provides similar local control to surgery with less long term morbidity therefore is preferable to surgery [3]. Similarly, radiotherapy alone (or combined with a neck dissection) for tonsillar carcinoma provides cure rates as good as surgery with a lower risk of complications [4].

Concurrent chemo-radiation rather than radiotherapy alone has been shown to significantly reduce the local recurrence rates and has an absolute benefit of 6.5% at 5 years in particular for patients with locally advanced disease [5]. This, however, increases toxicity so is only undertaken in patients with a good performance status. In addition the impact on functional morbidity has yet to be fully evaluated. If surgery is undertaken as the primary treatment, then post-operative radiotherapy may be offered as this improves outcome in node positive disease [6] or to high risk patients, post-operative chemoradiotherapy as this has been shown to improve survival further [7,8].

As non-surgical treatment has intensified in order to improve tumour control, and surgical techniques for resection and reconstruction have extended the surgical options, there is a clear need to consider the quality of life impact of these intensive often multi-modality schedules. The Liverpool group have been evaluating quality of life using the University of Washington Quality of Life questionnaire. They have recently published their work that augments patients' understanding of their treatment but it is based very much on the classical surgery and post-operative radiotherapy scenario [9].

Internationally, work is underway to develop readily usable core sets of questions based on the International Classification of Functioning, Disability and Health [10]. The Therapy Outcome Measure (TOM) scoring system uses these principles. Originally conceived by Enderby [11] it has been developed further particularly in Australia as a simple and reproducible way to assess the degree of dysphagia experienced by patients before, during and after treatment [12]. When considering outcome measures to use, Skeat and Perry stress the importance of the measure being practical to apply and that it presents an accurate picture of change [13].

This paper therefore evaluates tumour control and intermediate/late toxicity especially in relation to swallowing dysfunction in those patients with locally advanced oropharyngeal squamous cell carcinoma who have undergone either primary chemo-radiation or post-operative radiotherapy with or without concurrent chemotherapy. For the purposes of the study we used the TOM scoring system and specifically, the 'disability' descriptor that provided the best fit as a marker of outcome.

## Methods

All patients who were identified with locally advanced (stage 3/4) squamous cell oropharyngeal cancer irrespective of tumour type and who required radical radiotherapy were to be included in the series as long as they were treated with inverse-planned IMRT either as definitive or adjunctive therapy.

Initial patients were planned with the Varian Cadplan™ treatment planning system (TPS) (Varian Medical Systems, Palo Alto, CA), later patients with the Varian Eclipse TPS ™ both with the Helios™ inverse planning module.

All patients were treated as standard with contralateral parotid gland sparing (intended mean dose 26Gy)[14]. A seven field equi-spaced co-planar field arrangement was used doses in all cases.

Doses were specified to the mean of the PTV. Three dose levels were specified as appropriate. Dose level ONE: 65Gy/30#/6 weeks to the primary and involved nodes as definitive RT or post-operatively when margins were grossly involved or there was extra-capsular extension of nodes. Dose level TWO: 60/30#/6 weeks to areas considered at 'high-risk' in the definitive setting (e.g. level 3 nodes when level 2 nodes were positive) or the 'surgical bed' in post-operative cases. Dose level THREE: 54Gy/30#/6 weeks to the remainder of the neck for prophylactic irradiation (definitive or post-operative). The exception to this approach was in the post-operative setting where a few patients received an accelerated schedule i.e. 54Gy/34#/2.5 weeks as in the MRC Chartwel protocol [15].

Chemotherapy when used was delivered using a weekly schedule of 35 mg/m^2 ^for up to 6 cycles. The criteria for concurrent post-operative chemoradiotherapy was as per the EORTC study [8]. If the criteria relating to 'tumour factors' were met for concurrent chemotherapy but there was concern over the patients' level of fitness/suitability, cetuximab was considered as an alternative.

Toxicity was recorded prospectively using the RTOG scoring systems (for both acute and late effects). The Therapy Outcome Measure (TOM) score was recorded immediately prior to and following radiotherapy by either clinicians or the speech and language therapists (*table *1) and thereafter in follow up at 6 monthly intervals.

**Table 1:** TOM Score (Disability Domain)

0	Non oral feeding/supplements required to meet all hydration/nutritional needs
1	Non oral feeding/supplements required to meet hydration/nutritional needs. Consistently able to take practice amounts

2	Non oral feeding/supplements required to meet hydration/nutritional needs. Consistently able to take practice amounts

3	Consistently able to take modified consistencies using compensatory strategies. May require feeding supplements. May eat extremely slowly

4	Although eating & drinking is abnormal, it is good enough to meet nutritional requirements. No supplements. May avoid certain foods or eating situations

5	Functionally eating & drinking a normal diet

The insertion of prophylactic feeding tubes (RIGs and PEGs) were the preferred option in view of the predicted intensity of treatment.

Trismus, defined less than 35 mm mouth opening at incisors, was also assessed pre- and post- treatment.

Regular weights were obtained prior to, during and immediately post treatment and subsequently during routine follow up clinics.

The department protocol for follow-up by clinicians was weekly during treatment and once radiotherapy was complete until the acute reactions had subsided sufficiently. Patients were then reviewed initially after 3 weeks, then 6 weekly for the first 12-18 months and at less frequent intervals thereafter. Patients if free of disease and significant toxicity were offered discharge at 5 years.

## Results

24 patients were identified who received parotid-sparing IMRT between 1/2003 and 11/2007 and who had stage III/IV tumours (*table *2). No other patients were identified who had not been treated by a full inverse-planned approach during this period.

**Table 2:** Table of Patient Characteristics

Age	Median	53.5
	Range	44-74
Sex	Male	18
	Female	6

Primary site	Tonsil	11
	Base of tongue	10
	Soft palate	2
	Posterior pharyngeal wall	1

63% were T1/2, 33% T3/4 and 4% unclassified. 29% were N0, 8% N1, 59% N2 and 4% N3. 10/24 had primary RT, 14/24 had primary surgery.

Of those receiving primary RT, all bar one received a dose of 65Gy in 30# over 6 weeks (the other missed one fraction due to hospital admission). For those receiving post operative radiotherapy (n = 14), 9 (64%) received 65Gy/30#, 4 (29%) had 54Gy/36# and 1 (7%) received 64Gy/30# (*table *3).

**Table 3:** Table of Treatment Received

Primary radiotherapy (n = 10)	Neoadjuvant	Cisplatin/5FU	1
		Carboplatin/5FU	1
	Concurrent	Cisplatin	4
		Carboplatin	1
		Cetuximab	1
Post-operative radiotherapy (n = 14)	Concurrent	Cisplatin	4
		Carboplatin	1
		Cetuximab	1

In total 50% of patients received concurrent chemotherapy. In the majority of patients this was in the form of weekly cisplatin at a dose of 35 mg/m^2 ^and for an average of 5 cycles.

### Target Volumes

The mean combined planning target volume treated was 918 cm^3 ^(range 293.9 cm^3 ^- 2104.8 cm^3^). Averages for individual PTVs were PTV1 391.7 cm^3^, PTV2 311.7 cm^3 ^and PTV3 191.0 cm^3^. The figures take account that in some cases only two dose levels (and hence volumes were utilised). 8.3% (n = 2) patients received unilateral neck irradiation, the rest bilateral.

### Acute Toxicity

54% developed Grade 3 mucositis. No patients developed Grade 4 mucosal reactions. 12% required delay of radiotherapy or discontinuation but by no more than one fraction. 42% did not complete all 6 planned cycles of concurrent chemotherapy. One patient switched to carboplatin after 3 cycles of cisplatin as this was poorly tolerated but only received one further cycle.

25% (n = 6) of patients were admitted during treatment- one for an aspiration pneumonia, three for vomiting following concurrent chemo-radiation, one with constipation following chemo-radiation and one with diarrhoea and vomiting during radiotherapy alone.

The mean weight loss during radiotherapy was 9.26 kg (median 9 kg) which accounted for a mean of 12.75% of body weight (assessable in 19 patients). All but one patient had a gastrostomy (RIG) tube inserted prophylactically.

### Late Toxicity

**Figure 1:**
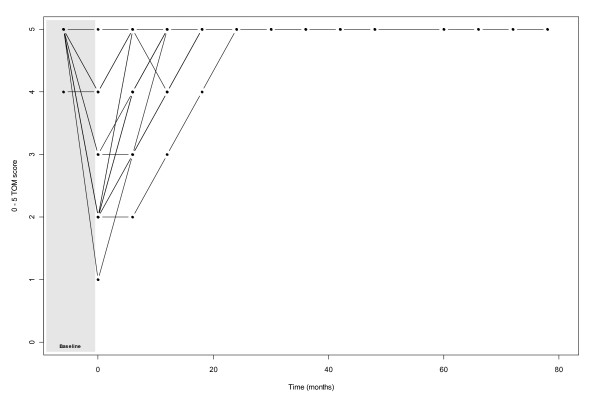
**Graph illustrating the recovery of TOM score over time for patients who recovered to at least their baseline swallowing function (n = 15) (Shaded area represents time during radiotherapy**)

**Figure 2:**
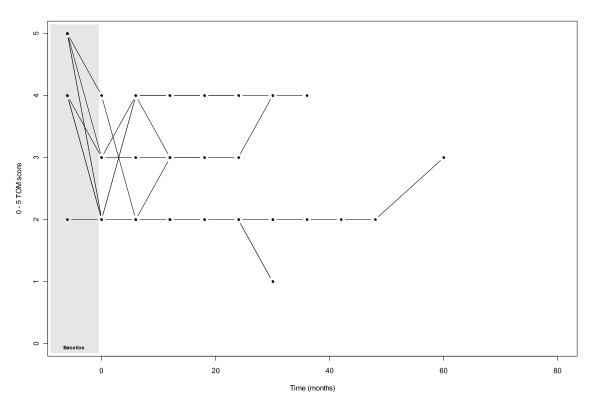
Graph illustrating the recovery of TOM score over time for patients who did not recover their baseline swallowing function (n = 9)

At mean follow-up of 20.7 months, 18% had returned to a normal diet (TOM score 5), 41% required some dietary modifications (TOM score 4), 18% required oral feeding supplements (TOM 3) and 23% were still dependent on nutritional support by means of RIGs (TOM scores of 0, 1 and 2). With longer follow-up (mean 37.1 months), TOM scores were 62.5%, 12.5%, 8% and 17% respectively. Of surviving patients at a mean follow up of 3.1 months post treatment, 2 still had RIG tubes in situ. For those patients whose swallowing function did recover post RT, it took on average 8.7 months to return to near normal eating function (i.e. TOM score of 4 or 5) (*figures *1*&*2*)*.

Of those patients who did not recover their baseline swallowing function, 56% (n = 5) were post operative. Of the patients who died within the follow up time, none regained their baseline swallowing function (n = 4).

There is long term data available for 83% of patients with regards to rates of trismus. 15% (n = 3) of these experienced trismus secondary to the radiotherapy itself. 10% (n = 2) had trismus which predated the radiotherapy. Trismus was actively managed with the use of regular spatula placement and/or the use of commercial devices (Therabite^®^).

One patient has developed a pharyngeal stricture and one patient only symptomatic osteoradionecrosis though residual disease was noted in the resected and reconstructed tissue. With a median follow-up of 31.6 months, 16.5% patients have died (n = 2 recurrence, n = 2 intercurrent illness).

### Survival

**Figure 3:**
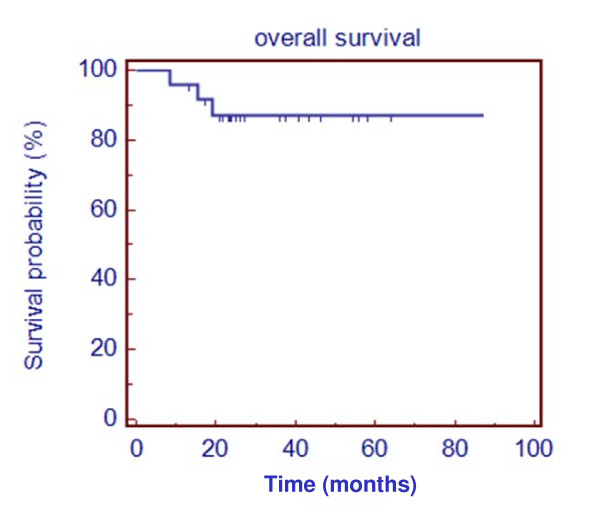
Kaplan Maier curve showing overall survival probability (%) against time (months)

The 2 year overall survival was 92% (n = 22), and disease specific survival 96% (n = 23) (*figure *3*)*.

## Discussion

The management of oropharyngeal cancer has undergone several treatment intensification strategies in order to improve disease control. In the main this has been through the use of concurrent chemotherapy but also targeting patients who historically might have been considered untreatable. At the same time, intensity-modulated radiotherapy (IMRT) has facilitated accurate dose delivery to carefully defined and delineated volumes. Uncertainty exists on the impact of intermediate doses delivered to normal tissues particularly concurrently with chemotherapy [16]. In addition, emerging data suggests an improved outcome for patients who are HPV positive and speculation that these patients might be suitable for treatment 'de-intensification' [17].

Our series has demonstrated excellent disease control for patients who historically have fared poorly. Mendenhall *et al. *in 2006 reported on one series of over 300 patients with base of tongue primaries [18]. In this series, local control rates at 5 years for Stage III, 82%; IVA, 87%; and IVB, 58%; the rates of absolute and cause-specific survival at 5 years were as follows: III, 66% and 77%; IVA, 67% and 84%; and IVB, 33% and 45%.

Assessments using subjective measures such as quality of life questionnaires, such as the University of Washington Quality of Life Scale [19] and objective assessments, for example, video fluoroscopy can be used to evaluate functional outcomes. The former could vary day to day dependent on patients' mood, and the latter is time consuming, invasive and expensive. The advantage of the TOM score as used here is that it can be performed swiftly at each and every review at the time or later potentially later, depending on the quality of the notes recorded. In considering the overall TOM score, we looked at both the ability to maintain nutrition orally and the amount of effort and compensatory techniques employed to achieve this.

It is standard practice within our department for patients receiving oropharyngeal and neck radiotherapy to have percutaneous enteral nutrition. Enteral nutrition has been shown to prevent weight loss, dehydration, nutritional deficiencies, treatment interruptions and hospitalisations along with providing an improved quality of life [20].

In one study of patients with pharyngeal squamous cell carcinoma who received radical radiotherapy in 1998-2002, 83% had some degree of dysphagia [21]. Aspiration was recorded in 18%. Fibreoptic evaluation of swallowing and quality of life questionnaires were used to assess patients after treatment. In comparison, 4% (n = 1) in our series developed any complications from aspiration. There may be an element of silent aspiration in this cohort but we also took a proactive approach to swallowing dysfunction during and post-treatment. FEES was used to evaluate any patients we had any concerns about in order to inform our advise and to facilitate and practice compensatory and rehabilitative techniques at appropriate times.

Rosenthal noted that 20% of patients post radiotherapy required long term enteral feeding [22] compared with our figure of 8%. Mekhail et al. found the average time for a PEG feeding tube to be in situ was 6.5 months [23]. Our data compares favourably with this at an average of 3.1 months post completion of radiotherapy.

Rademaker *et al*, 2003 indicated that it took approximately 1 year to recover eating ability to near normal level [24]. In our series recovery was somewhat shorter at an average of 8.7 months. We routinely see our patients before, during and after treatment in order to assist with swallow rehabilitation and encourage them back towards attempting oral foods. A monthly joint nutrition/SLT clinic is held where appropriate advice and decisions about adequacy of enteral feeds can be actioned as oral intake increases.

When treating oropharyngeal cancers, radiotherapy using a large radiation portal field (>11 cm) has been shown to have a significant impact (p < 0.001) on swallowing dysfunction [25]. The work spans a pre- and post-IMRT era. Previous studies have confirmed the significant decrease in volume of tissue irradiated when using IMRT techniques compared with standard non-conformal plans. Some studies have shown that irradiation of the pharyngeal constrictor muscles play a role in resulting dysphagia. One study of 88 patients with oropharyngeal carcinomata treated with radiotherapy with or without concurrent chemotherapy suggested a probability of dysphagia of 19% with every additional 10Gy to the constrictor muscles [26]. However, a further smaller study by Bhide et al. [27] did not find a statistically significant relationship between radiation dose to the pharyngeal constrictors and dysphagia at 1 year post chemo-radiation for head and neck cancer. Further investigation in this area is warranted. In our series, no specific attempt was made to spare the pharyngeal constrictors. In part this was because they were not well visualised (planning MRIs have since been adopted), there is no consensus yet on their anatomical definition and due to a policy that still exists of 'anatomical' rather than 'volumetric' primary target inclusion.

### Trismus

Trismus is has been noted to be exceedingly common both post conventional radiotherapy and IMRT to head and neck cancer, with one study suggesting a rate of 45% [28]. The rate of trismus in this series is low at 15% and may reflect the pro-active approach in its prevention. Previous studies have shown an increase in mandibular dysfunction as radiation dose to the pterygoids is increased [29]. With the increased conformality that IMRT is able to offer, similar to the potential benefit of dose constraint to the constrictor muscles, there may also be some benefit to sparing of the contralateral pterygoid muscles. Although in this series there was no specific dose constraint on these muscles and a 'whole-organ'approach was used (i.e. the whole oropharynx), the contralateral pterygoid muscles were generally excluded from the primary CTV.

### Weight Loss

Median weight loss during treatment previously has been recorded as 18 lbs (8.16 kg) by Nguyen et al. [30]. This is comparable with our median weight loss of 9.26 kg (20 lbs) despite proactive management. In the Nguyen series, weight loss of more than 20% during treatment predicted for poor outcome with regards to not regaining baseline swallowing function (p = 0.0002). This highlights the importance of multidisciplinary team assessments during radiotherapy and the necessity for feeding supplements throughout. In addition, weight loss can mean a change in body contour in the treated area affecting set-up and coverage of the clinical target volumes. We found no evidence of this being an issue in our series. We are currently evaluating the role of weekly cone-beam KV CT imaging to assess for set-up errors (in addition to daily KV-KV planar imaging) and the potential for adaptive planning based on tumour response and/or changes in body profile. The potential value has been demonstrated elsewhere [31].

### Admission Rates

Admission rates, although high, are in keeping with other published series. Brady et al. [32] looked at admission rates for patients undergoing radiotherapy with or without concurrent chemotherapy for head and neck primaries and reported this to be 28% during treatment.

## Conclusions

Excellent disease control with intensified schedules of radiotherapy with IMRT has been achieved in this patient population with locally advanced oropharyngeal SCC. Intermediate toxicity is significant but with longer follow-up, dysphagia continues to improve with 75% of patients not requiring any form of enteral or oral supplementation.

Social and emotional aspects of eating and drinking difficulties are important factors to consider alongside the initial physical recovery of swallow function and the ability to maintain nutrition orally. Ongoing informed support and appropriate and timely encouragement from clinical staff, carers and volunteers in addressing these issues contributes greatly towards long-term self management and survivorship. The TOM scoring system, as used here, provided a useful means of evaluating functional recovery of swallowing.

The data lends support to such intensive treatment approaches to achieve maximal cure rates but is reliant on good multi-disciplinary working and appropriate patient selection.

## Competing interests

The authors confirm that they have no competing interests.

## Authors' contributions

CJI and CDS wrote the initial draft and revised the draft after receipt of comments from KY, VC, CD and AF. All authors approved the final version for publication. CDS is the guarantor.
